# Dental Treatment of a Child with Pallister-Killian Syndrome

**DOI:** 10.1155/2016/4130961

**Published:** 2016-02-21

**Authors:** Serhan Didinen, Didem Atabek, Gülay Kip, Aslı Patır Münevveroğlu, Özlem Tulunoğlu

**Affiliations:** ^1^Department of Pediatric Dentistry, Faculty of Dentistry, Istanbul Medipol University, Turkey; ^2^Department of Pediatric Dentistry, Faculty of Dentistry, Gazi University, Turkey; ^3^Department of Anesthesia and Reanimation, Faculty of Medicine, Gazi University, Turkey; ^4^Department of Pediatric Dentistry, School of Dental Medicine, Case Western Reserve University, Cleveland, OH, USA

## Abstract

The Pallister-Killian syndrome (PKS) is an extremely rare genetic disorder with an incidence estimated around 1/25000. PKS is a multiple congenital anomaly deficit syndrome caused by mosaic tissue limited tetrasomy for chromosome 12p. The presented report is the first confirmed case with PKS in Turkey. This report focuses on the orofacial clinical manifestations of an 6-year-old boy with PKS who was referred to the Department of Paediatric Dentistry clinic, Gazi University. It has been learned that the PKS was diagnosed 1 year after birth. Due to intellectual disability, it was decided to make the dental treatments under moderate sedation. Although significant tongue thrust and anterior open bite were determined, any oral appliances could not be applied because of the 2 epilepsy seizures in the last 2 years. The aim was to treat decayed teeth and set good oral hygiene in the patient's mouth. Still, there is a probability for epilepsy seizures. If epileptic seizures stop permanently, we can apply an oral appliance to block tongue thrust. The patient is now under control. In cases of systemic and oral findings such as PKS, conducting medical and dental approaches together will increase the life quality of patients.

## 1. Background

The Pallister-Killian syndrome (PKS) is an extremely rare genetic disorder with an incidence estimated around 1/25000. PKS is a multiple congenital anomaly complex mosaic genetic duplication syndrome caused by mosaic tissue limited tetrasomy for chromosome 12p. It is defined as a mosaic condition as a result of not all cells having an extra chromosome [1, 2]. When genetic changes occur somatically, the individual exhibits cells with at least two different genotypes, and this state is known as mosaicism [3]. Somatic mosaicism is defined as the presence of two or more populations of cells with different genotypes in an individual who has developed from a single fertilized egg [2].

PKS has variable clinical manifestations and the syndrome may affect different organs and body systems. Although none of the features is pathognomonic for this chromosomal disorder, the more consistent anomalies include craniofacial dysmorphism (prominent forehead, sparse anterior scalp hair, flat occiput, hypertelorism, short nose with anteverted nostrils, flat nasal bridge, and malformed ears), short neck, limb deformities, pigmentary skin anomalies, and nail hypoplasia [4–6]. Progressive psychomotor developmental delay, severe hypotonia, deafness, and seizures become more prominent with age. In contrast, in perinatally diagnosed cases, a much higher incidence of internal organ anomalies, like congenital diaphragmatic hernia, cardiovascular anomalies, and anorectal anomalies, is noted [7]. This age-dependent phenotype expression is another characteristic of PKS [4].

Frequency of seizures, age of onset, typical seizure types, response to treatment, and prognosis of epilepsy are hardly known in PKS [8]. Epileptic seizure prevalence in PKS is in the vicinity of 42–59% as shown by case series and meta-analysis studies [9, 10].

This report provides the dental treatment of a 6-year-old boy with two sessions of deep sedation. To our knowledge, the presented case is the first confirmed dental patient with PKS in Turkey.

## 2. Procedure

**Figure 1:**
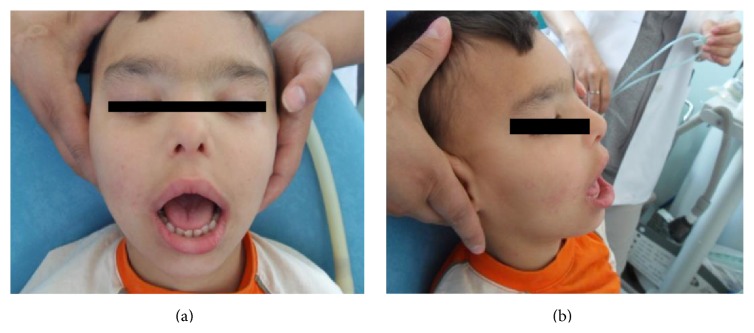
Orofacial clinical manifestations of a 6-year-old boy with PKS.

This report focuses on the orofacial clinical manifestations of a 6-year-old boy with PKS who was referred to the Department of Pediatric Dentistry clinic, Faculty of Dentistry, Gazi University (Figures 1(a) and 1(b)). It has been learned that the PKS was diagnosed 1 year after birth.

Orbital hypertelorism and esophoria, bitemporal alopecia, bilateral hearing loss, open posture of mouth, prominent and large forehead, flat nose bridge, and short nose are observed extraorally.

**Figure 2:**
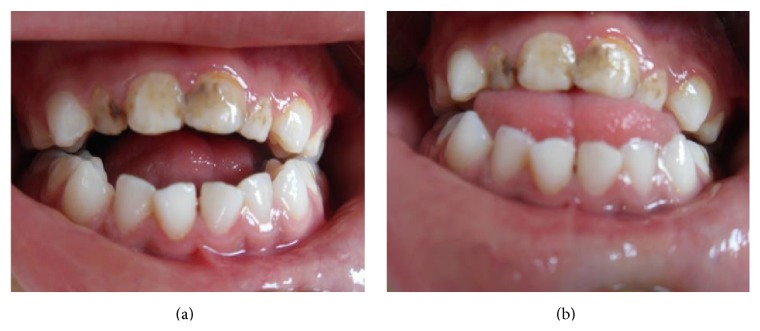
Oral findings related to PKS: oral breathing, anterior open bite, tongue thrust, mandibular prognathism, and macroglossia.

**Figure 3:**
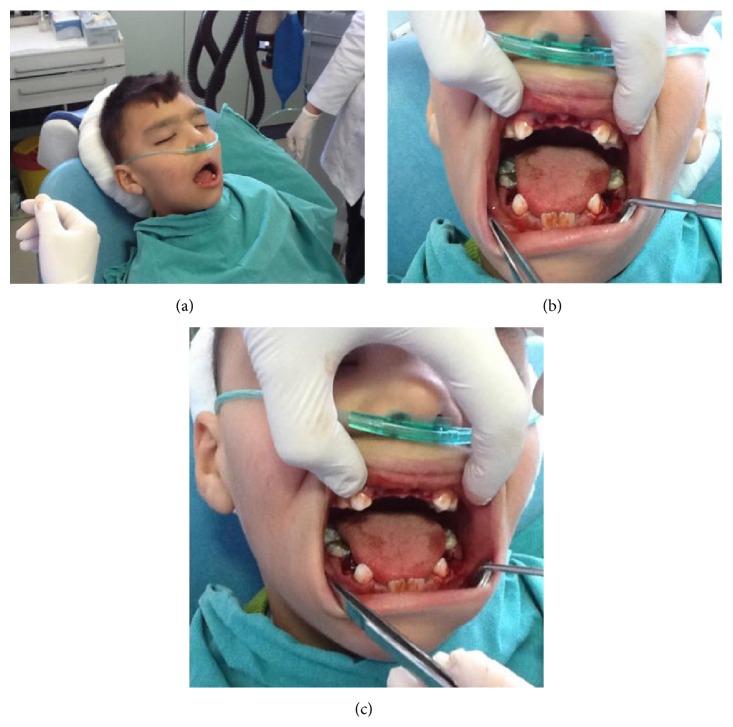
Considering the patient's mental retardation and lack of cooperation on dental chair, it was decided to make the dental treatments under moderate sedation.

During the clinical examination, some oral findings related to PKS were diagnosed as follows (Figures 2(a) and 2(b)): oral breathing, anterior open bite, tongue thrust, mandibular prognathism, and macroglossia. It was observed that there are shallow caries in teeth #55 and #65 and profound caries in teeth #51, #52, #61, #62, #36, #46, #74, #75, #84, and #85. Considering the patient's mental retardation and lack of cooperation on dental chair, it was decided to make the dental treatments under moderate sedation. During the first session of sedation, teeth #51, #52, #61, #62, #74, #75, and #84 were extracted and #85, #36, and #46 were restored with amalgam restoration (Cavex, Haarlem, Netherlands) (Figures 3(a), 3(b), and 3(c)).

**Figure 4:**
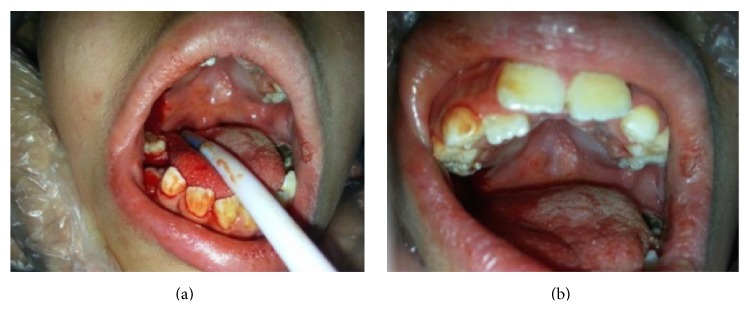
Newly decayed teeth were observed and a new treatment plan was established for second session of moderate sedation.

After the dental treatments under sedation, the patient's family were advised to clean their child's teeth regularly and visit our clinic every 6 months, for follow-ups. However, the patient returned to the clinic 3 years after treatment again with pain in his teeth. Due to lack of routine controls, newly decayed teeth were observed and a new treatment plan was established for second session of moderate sedation. Under sedation, teeth #55 and #46 were restored with composite, resin (Tetric-N-Ceram, Ivoclar Vivadent, Schaan, Liechtenstein) (Figures 4(a) and 4(b)).

After this session, the patient's parents changed their phone number without declaration. Also, they have not visited our clinic again, for follow-up controls or any other complaints.

## 3. Discussion

Severe to profound developmental delay is observed in most individuals with PKS, although there have been an increasing number of mildly affected individuals with PKS reported [11]. Average ages for achieving motor milestones in PKS include 10.8 months (rolling), 21.2 months (sitting independently), and 38.8 months (walking). Although there are many individuals with PKS who do not attain speech, initiation of speech often starts at 36 months [12]. Repetitive hand and body movements were frequently noticed (75%), and this condition makes the dental treatment impossible under full-consciousness situation [2]. General anesthesia and deep sedation was observed as an effective dental treatment option for these types of patients who have involuntary movements. Also, hear loss and cataract are other rough factors to communicate with the patient.

Routine dental procedures can be successfully performed under sufficient precautions [13]. Treatment of oral habits in children with special care poses a challenge for dentists. Reminder therapy and use of a reward system should be attempted prior to the placement of any dental appliance [14]. Dentists should use corrective intraoral appliances to treat thumb-sucking if the habit is chronic, if the child above 4 years old, and if the child requests help in stopping the habit [15]. Although the use of fixed appliances is indicated in a child with epilepsy, due to the risk of dislodgement and potential airway obstruction [16], the neurologist advised us not to apply a fixed or removable oral appliance, till seizures totally stop.

Mouth breathing and tongue thrusting can be counted as factors for future caries and malocclusion [17]. Chronic mouth breathing occurs with the obstruction of the nasal airway which is caused by chronic allergy and the excessive lymphoid tissue proliferation [18]. Mouth breathing was shown as an etiological factor for tongue thrusting and anterior dental open bite in current studies [19]. When the malocclusion remains for a long period of time, the tongue fills the oral cavity and makes lips lose their tonus [20–22]. The presented case exhibits mouth opening since he was born and open bite due to this habit.

## 4. Conclusion

Pallister-Killian syndrome is an extremely rare syndrome and dentists are unfamiliar with how to manage it. Dentists should warn the parents about preventive issues and regular dental visit to save children from caries. However, if the patient is seen in a bad oral situation, deep sedation is a beneficial way to treat all teeth in one session.

## References

[1] Murakami M., Iwasa T., Takahashi Y., Morine M. (2011). Amniocentesis can be useful during the third trimester of pregnancy for antenatal diagnosis of Pallister-Killian syndrome: a case report. *Clinical and Experimental Obstetrics and Gynecology*.

[2] Izumi K., Krantz I. D. (2014). Pallister-Killian syndrome. *American Journal of Medical Genetics. Part C: Seminars in Medical Genetics*.

[3] Spinner N. B., Conlin L. K. (2014). Mosaicism and clinical genetics. *American Journal of Medical Genetics C*.

[4] Baglaj M., King J., Carachi R. (2008). Pallister-Killian syndrome: a report of 2 cases and review of its surgical aspects. *Journal of Pediatric Surgery*.

[5] Reynolds J. F., Daniel A., Kelly T. E. (1987). Isochromosome 12p mosaicism (Pallister mosaic aneuploidy or Pallister-Killian syndrome): report of 11 cases. *American Journal of Medical Genetics*.

[6] Horneff G., Majewski F., Hildebrand B., Voit T., Lenard H.-G. (1993). Pallister-Killian syndrome in older children and adolescents. *Pediatric Neurology*.

[7] Mowery-Rushton P. A., Stadler M. P., Kochmar S. J., McPherson E., Surti U., Hogge W. A. (1997). The use of interphase FISH for prenatal diagnosis of Pallister-Killian syndrome. *Prenatal Diagnosis*.

[8] Candee M. S., Carey J. C., Krantz I. D., Filloux F. M. (2012). Seizure characteristics in Pallister-Killian syndrome. *American Journal of Medical Genetics Part A*.

[9] Stalker H. J., Gray B. A., Bent-Williams A., Zori R. T. (2006). High cognitive functioning and behavioral phenotype in Pallister-Killian syndrome. *American Journal of Medical Genetics—Part A*.

[10] Bielanska M. M., Khalifa M. M., Duncan A. M. V. (1996). Pallister-Killian syndrome: a mild case diagnosed by fluorescence in situ hybridization. Review of the literature and expansion of the phenotype. *American Journal of Medical Genetics*.

[11] Kostanecka A., Close L. B., Izumi K., Krantz I. D., Pipan M. (2012). Developmental and behavioral characteristics of individuals with Pallister-Killian syndrome. *American Journal of Medical Genetics Part A*.

[12] Wilkens A., Liu H., Park K. (2012). Novel clinical manifestations in Pallister-Killian syndrome: comprehensive evaluation of 59 affected individuals and review of previously reported cases. *American Journal of Medical Genetics—Part A*.

[13] Jacobsen P. L., Eden O. (2008). Epilepsy and the dental management of the epileptic patient. *Journal of Contemporary Dental Practice*.

[14] Greenleaf S., Mink J. (2003). A retrospective study of the use of the Bluegrass appliance in the cessation of thumb habits. *Pediatric Dentistry*.

[15] Chhabra N., Chhabra A., Bansal S. (2012). An innovative approach to cessation of thumb-sucking in a child with epilepsy: a case report. *Special Care in Dentistry*.

[16] Vorkas C. K., Gopinathan M. K., Singh A., Devinsky O., Lin L. M., Rosenberg P. A. (2008). Epilepsy and dental procedures. A review. *The New York State Dental Journal*.

[17] Indira M. D., Dhull K. S., Nandlal B., Praveen Kumar P. S., Dhull R. S. (2014). Biological restoration in pediatric dentistry: a brief insight. *International Journal of Clinical Pediatric Dentistry*.

[18] Watson W. G. (1981). Open-bite-a multifactorial event. *American Journal of Orthodontics*.

[19] Greenlee G. M., Huang G. J., Chen S. S.-H., Chen J., Koepsell T., Hujoel P. (2011). Stability of treatment for anterior open-bite malocclusion: a meta-analysis. *American Journal of Orthodontics and Dentofacial Orthopedics*.

[20] Haralur S. B., Al-Qahtani A. S. (2013). Replacement of missing anterior teeth in a patient with chronic mouth breathing and tongue thrusting. *Case Reports in Dentistry*.

[21] Ahmad N. E., Sanders A. E., Sheats R., Brame J. L., Essick G. K. (2013). Obstructive sleep apnea in association with periodontitis: a case-control study. *Journal of Dental Hygiene*.

[22] Pennel B. M., Keagle J. G. (1977). Predisposing factors in the etiology of chronic inflammatory periodontal disease. *Journal of Periodontology*.

